# Cases of Poisoning by Belladonna

**Published:** 1845-11

**Authors:** H. M. Gray

**Affiliations:** New York, Physician to the N. Y. City Dispensary


### SELECTIONS

FROM

### AMERICAN AND FOREIGN JOURNALS.

---

*Cases of Poisoning by Belladonna.* By H. M. Gray, M. D., of New York, Physician to the N. Y. City Dispensary.—

Case I.—The subject of the first case was a child between two and three years of age, who had been suddenly seized with alarming symptoms. Suspecting the patient to be laboring under the effects of poison, an inquiry was instituted to ascertain the fact. The mother found in the possession of the child a box from which she had been eating, as shown by the stains upon her lips and fingers. It proved to be the extract of atropa belladonna, which had been used a short time previously by one of the family, in a neuralgic affection. The amount swallowed, as near as could be ascertained at the time, was from eight to twelve grains. The following symptoms presented themselves upon seeing the case some thirty or fifty minutes after the drug had been taken into the stomach.

The expression of the countenance was that of a person in *terror*—*pupils widely dilated* and *immovable*—the *tunica conjunctiva highly injected*, and the *whole eye prominent and preternaturally brilliant*. The face, upper extremities, and trunk of the body exhibited a diffuse scarlet efflorescence studded with innumerable papillæ, very closely resembling the rash of scarlatina; the eruption terminated abruptly at the wrists and flexure of the thighs, the rest of the body retaining the natural color. Skin hot and dry, and pulse much increased in force and frequency.

The patient's manner was apoplectic, respiration anxious, and attended with the brazen, stridulous sound of croup. A constant but unsuccessful effort at deglutition was observable, and at every renewal of the attempt the muscles of the throat and pharynx would be thrown into violent spasmodic action. Severe engorgement of the venous trunks was also present. This state of partial coma was alternated by paroxysms of uncontrollable tendency to motion and rapid automatic movement, attended with convulsive laughter. No well marked convulsions made their apperance; although during the brief periods of sleep into which the patient would fall, a slight subsultus of the muscles of the face and extremities was noticed.

The treatment was that ordinarily pursued in similar cases. The lower extremities were immersed in a mustard bath, while water and pounded ice were applied to the head. An active emetic was immediately administered whose operation was induced by the application of local stimulants to the epigastric region, and the free use of warm diluents. The matter vomited contained several portions of the drug in a partially dissolved state. As soon as free emesis had been procured, a strong decoction of coffee was ordered, to combat the soporific effect of the poison, alternated with diluted aqua ammonia for the purpose of decomposing any of the belladonna that might still remain in the stomach. The diuretic effect of the atropa now began to be experienced, the patient evacuating an enormous quantity of limpid urine. The alarming symptoms passed off in about three hours after the commencement of the treatment, and the child soon recovered with the exception of a moderate diarrhœa, and a slight enlargement of the pupil. The eruption had entirely faded.

Case II.—I will relate another case, concerning which I can speak somewhat experimentally, it having occurred in my own person. It is of interest only inasmuch as I was able to note accurately my own sensations during the action of the narcotic. This is an interesting point in the investigation of cases of poisoning, and one about which little can be known, as patients are generally too much occupied with their fears or actual sufferings, to be able to impart much knowledge of their sensations. Although pretty thoroughly narcotized, I watched with some curiosity and care the phenomena induced by belladonna. I had taken an unwarrantably large dose of the article in question, to quiet the pain of a severe neuralgic toothache; not finding any relief, I repeated it in the course of ten or fifteen minutes, swallowing in all some eight or ten grains. About an hour after the last dose had been taken, the medicine began to induce its specific symptoms in the following order. First, vertigo, increasing to such an extent as to render it impossible to walk without staggering. The dizziness which was at first transient, soon became continued, and very severe. Now came on the affection of the eyesight, every object growing dim, as though a cloud were between the eye and it. Sometimes objects appeared double, and with an undulating motion passed before the eye. I observed that by a strong effort of the will, a concentration of the nervous power, this paralysis of the retina might for a moment be combatted; but only to return with greater severity when the mental effort had been succeeded by its corresponding relaxation. The appearances of the eye were much the same as those mentioned in the former case, viz: pupil *immovably dilated;* eye *prominent*, *dry*, and *exceedingly brilliant.* The eonjunctival vessels were fully injected. There was *total absence of lacrymation*; and *motion* was *attended with a sense of dryness and stiffness.* The face was *red* and *turgid*, and the *temperature* and *color* of the surface *considerably augmented.* Pulse *full*, and about 120 to 30.

The feeling in the head was that of *violent congestion*, *a full*, *tense*, *throbbing state* of the *cerebral vessels*, identically the same sensation as would be produced by a ligature thrown about the neck; and impeding the return of the venous circulation. The peculiar state of the throat next excited attention. The *tongue*, *mouth* and *fauces were devoid of moisture, as if they had been composed of burnt shoe leather.* The *secrecretions* of the *glands* of the *mouth*, *and the saliva*, *were entirely suspended.* A draught of water, instead of giving relief, seemed only to increase the unctuous, clammy state of the mucuous membrane. About the bag of the pharynx this sensation was most distressing. It induced a *constant attempt at deglutition*, and finally excited *suffocation*, *spasms of the fauces and glottis*, renewed at every effort to swallow. A *little saliva*, *white*, and *round* like a *ball of cotton*, would now and then be evacuated.

The slight delirium that followed the action of the narcotic, was of a strange, yet not unpleasant kind. I wished to be in constant motion, and it certainly afforded me an infinite deal of satisfaction to be able to walk up and down. The intellectual operations at times were very vived. Thoughts came and went, and ludicrous and fantastic spectacles were always uppermost in my mind. I was conscious that my language and gesticulations were extravagant, yet I had neither power nor will to do otherwise than I did; and notwithstanding my bodily malaise, the mind was in a state of delightful exhilaration.

The treatment was very simple; cold douche to the head. and an emetic, soon destroyed the dominion of poison.

In this case as in the other, I found some diffculty in provoking the operation of an emetic, owing to the insensible condition of the stomach. After vomiting, the disposition to sleep became very urgent. Strong coffee, however, counteracted this tendency.

One other fact with reference to the effects produced by Belladonna is worthy of note, viz its *tremendous diuretic power.* I have observed that it does not seem to reach the kidneys, until it has been some time in the stomach, and has exerted its specific influence upon the brain. But its power over the secretion of urine seems to be very great. I am confident I passed in the course of an hour three pints of urine, accompanied with a slight strangury at the neck of the bladder.

The nature, properties and effects of atropa belladonna are so well known, that I need not enlarge this paper by alluding to this subject. As there appears to be no positive antidote to its poisonous effects, the treatment must be based upon general principles.

Reasoning from the effects it produces upon a healthy body, its indications in disease become evident. From its property of dilating the pupil it becomes an indispensable in the treatment of iritis, and incipient cataract. In many other affections of the eye, it is a remedy of essential importance. Its effects in painful ulcerations—in pertussis—in neuralgia, and in a host of nervous maladies, are universally recognised.

From its effects as observed in my own case, I am induced to believe that it may become an important remedy in diseases, in the treatment of which, at present, it is but little used.

We have seen the entire suppression of the lachrymal and salivary discharge which it induces. Now in stomatitis, either aphthous, or mercurial attended with profuse discharge of saliva, and in epiphora of the lachrymal sac, I cannot see why it is not the very remedy indicated. In irritability of the eye, more particularly of the retina, its quieting effect would be of immense service. In the cachectic dropsies, attended with great irritability of the system, it might also be of use. In anæmia of the brain its congestive powers might be used with evident advantage. The catalogue might be extended, but it is sufficient for purposes of illustration. At all events whether we adopt the principle, "similia similibus curantur"—or "contraria contrariis curantur," the only effective mode of discovering the indications of disease, and the proper application of therapeutic agents to meet or combat these indications is by experiments upon the healthy, as well as the diseased body, carefully watching the effects produced in each.—*N. Y. Jour. Med.*

